# Supplementary material for: Mucin Expression Profiles in Ulcerative Colitis: New Insights on the Histological Mucosal Healing
Source: Int J Mol Sci. 2024 Feb 3;25(3):1858. doi: 10.3390/ijms25031858 (PMC10855303; doi:10.3390/ijms25031858)
Supplement: Supplementary file 1 [file ijms-25-01858-s001.zip › ijms-2842599-supplementary.pdf]

Mucin expression profiles in Ulcerative Colitis: new insights on the histological mucosal healing

Leoncini Giuseppe, MD[1], Cari Luigi PhD[2], Ronchetti Simona PhD[2], Donato Francesco MD[3], Caruso Laura[4], Calafà Cristina[4], Villanacci Vincenzo MD[5]

[1] First Pathology Division, Department of Pathology and Laboratory Medicine, Fondazione IRCCS Istituto Nazionale dei Tumori, Milan, Italy.
[2] Pharmacology Division, Department of Medicine and Surgery, University of Perugia, Perugia, Italy
[3] Unit of Hygiene, Epidemiology and Public Health, University of Brescia, Brescia, Italy
[4] Pathology Unit, Department of Pathology and Laboratory Medicine, Desenzano del Garda Hospital, ASST del Garda, Brescia, Italy
[5] Institute of Pathology, ASST Spedali Civili, Brescia, Italy

Supplementary Materials

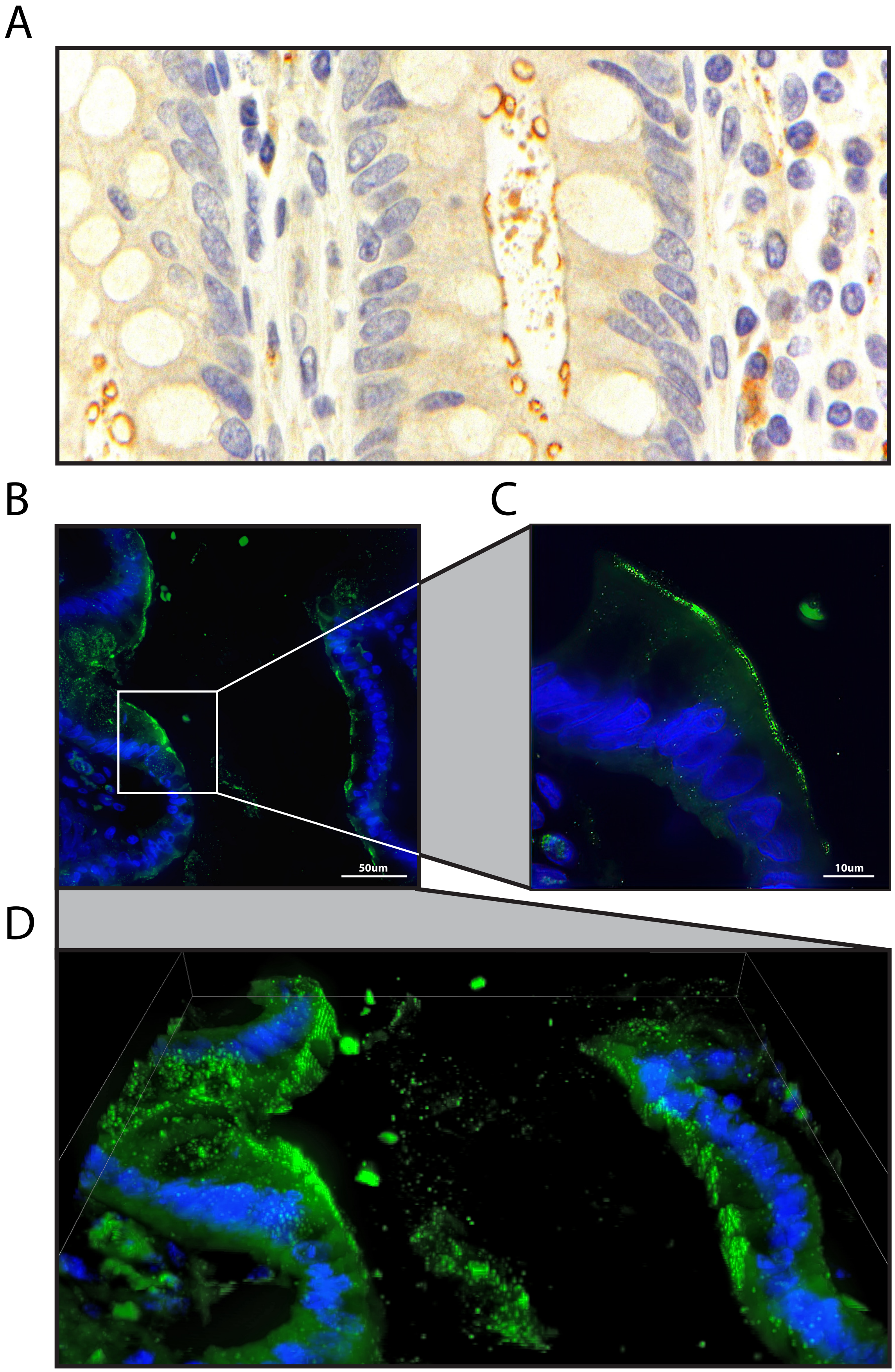

Figure S1. IHC and IF Staining of MUC15

The *membranous* (m) expression pattern of MUC15 appears faint in immunohistochemical analysis (**A**) but is clearly visible in confocal immunofluorescence analysis. Sixty-six focal planes (Z-range 19.5µm, Z-step 0.30µm) were acquired, and the maximum intensity projection (MIP) is presented in panel **B**. Details of the membranous MUC15 expression pattern are depicted in panel **C** through a deconvoluted cropped image. Panel **D** showcases a confocal 3D reconstruction of the same field. IHC magnification: x20; IF magnification: x60

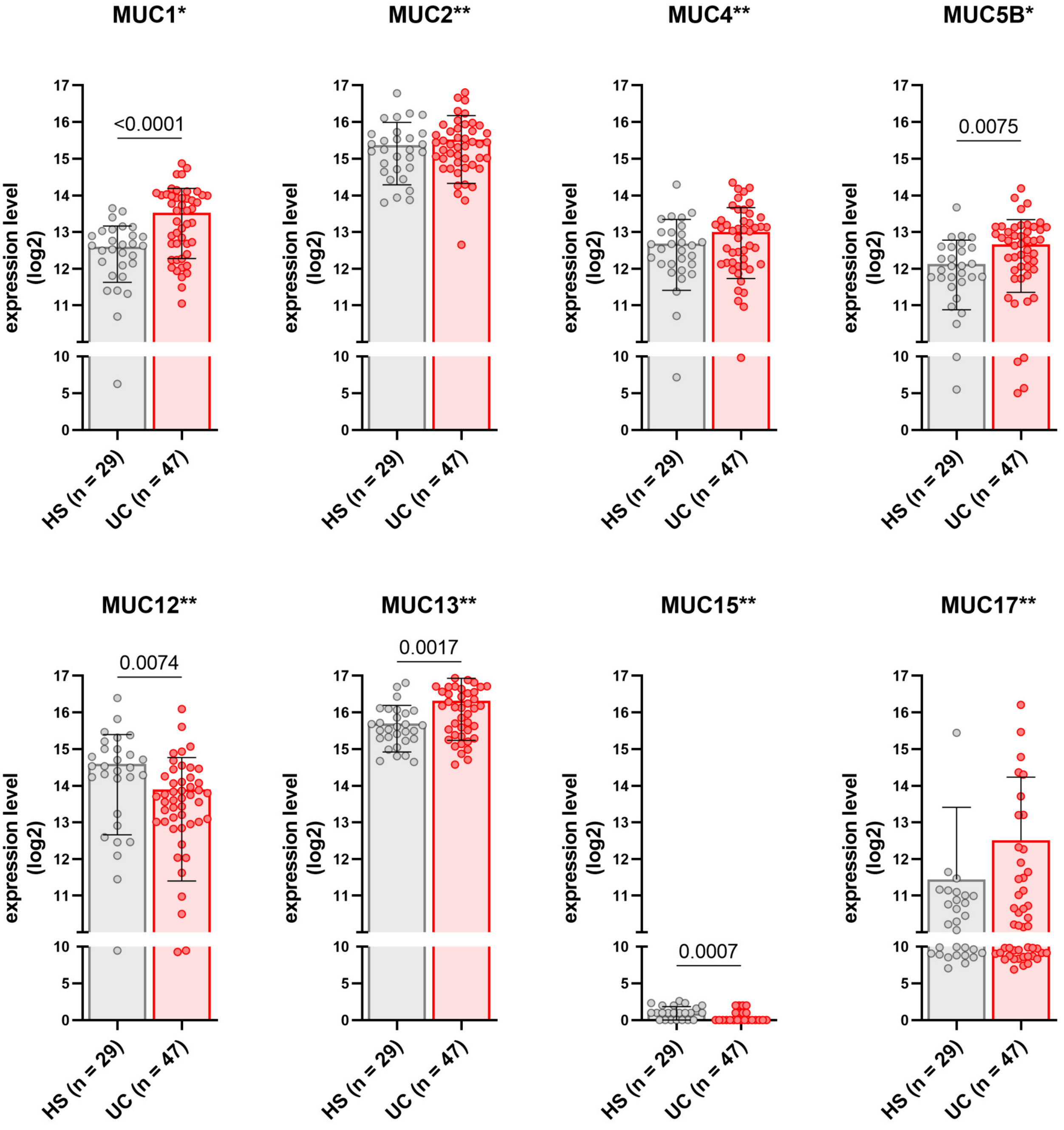

Figure S2. Transcriptional mucin expression profile
Transcriptomics data were retrieved from the *Inflammatory Bowel Disease Multi'omics Database* (IBDMDB). Mucin expression levels were compared between healthy donors (gray, n = 27) and UC patients (red, n = 47). The data are presented using a histogram, where each dot represents an individual patient; expression values ± SD are displayed on a log2 scale. P-values < 0.05 were considered statistically significant; *unpaired t-test (Welch); ** Mann-Whitney test.

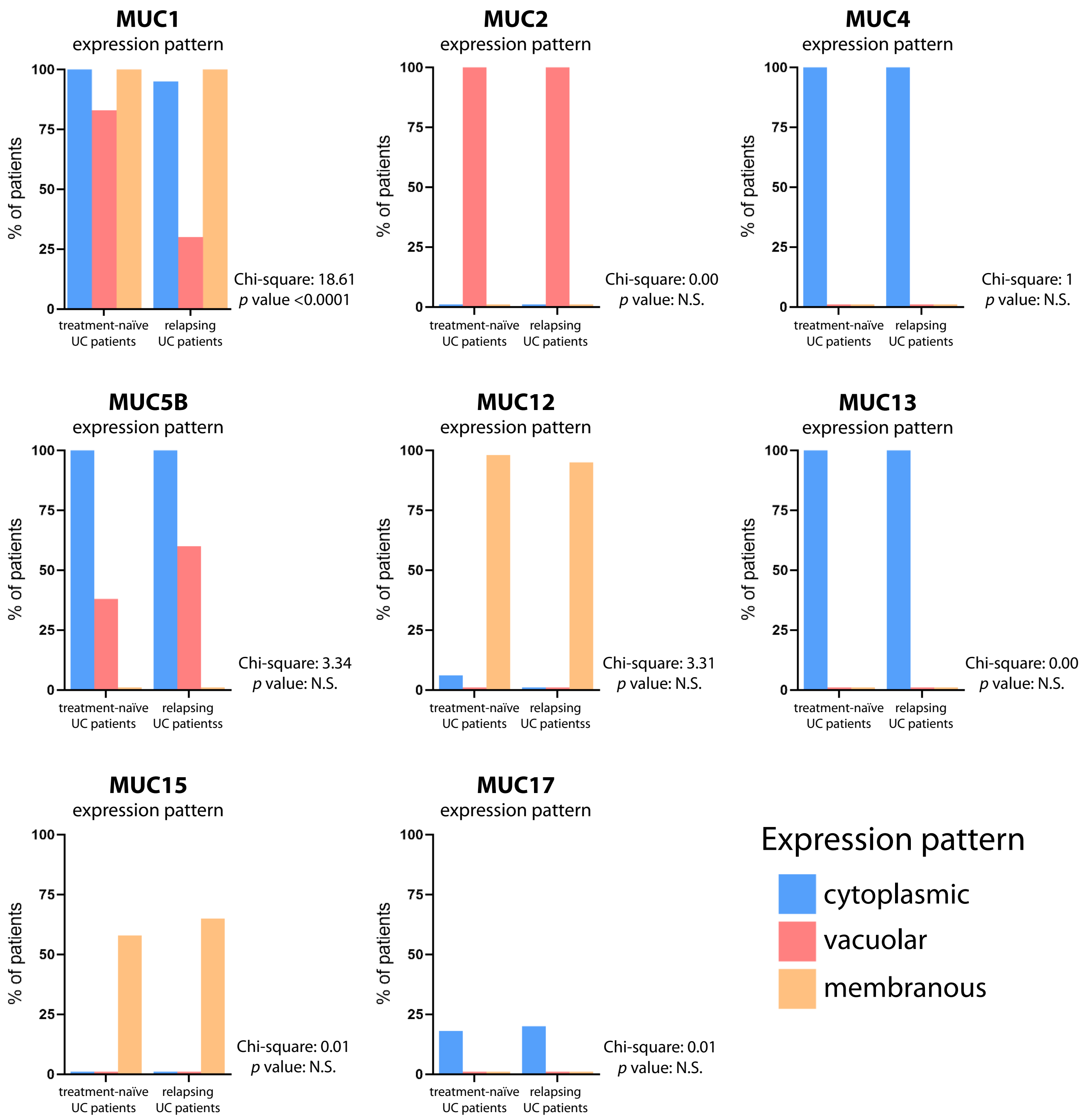

Figure S3. Mucin expression pattern comparison between treatment-naïve and relapsing UC patients

The graphs depict the mucin expression patterns in both the treatment-naïve and relapsing UC patients. The percentage of patients displaying cytoplasmic (blue bar), vacuolar (red bar), and membranous (yellow bar) expression patterns is reported. The contingency test (Chi-square test) was utilized to assess the statistical significance of agreement in mucin expression patterns, with a significance level set at $p < 0.05$

A

Factors predicted within a dissimilarity margin less or equal than 5 % :

GR-beta [T01920]	RXR-alpha [T01345]	C/EBPbeta [T00581]	YY1 [T00915]
XBP-1 [T00902]	c-Myb [T00137]	STAT4 [T01577]	c-Ets-1 [T00112]
SRY [T00997]	TCF-4E [T02878]	GR [T05076]	NF-Y [T00150]
Sp1 [T00759]	T3R-beta1 [T00851]	GCF [T00320]	PPAR-alpha:RXR-alpha [T05221]
TFIID [T00820]	NF-AT1 [T01948]	c-Myc [T00140]	USF1 [T00874]
STAT1beta [T01573]	IRF-1 [T00423]	GATA-2 [T00308]	MAZ [T00490]
PXR-1:RXR-alpha [T05671]	VDR [T00885]	NF-1 [T00539]	C/EBPalpha [T00105]
Pax-5 [T00070]	GR-alpha [T00337]	TFII-I [T00824]	AP-2alphaA [T00035]
c-Ets-2 [T00113]	IRF-2 [T01491]	p53 [T00671]	GATA-1 [T00306]
ENKTF-1 [T00255]	FOXP3 [T04280]	PR B [T00696]	PR A [T01661]
CREB [T00163]	ATF3 [T01313]	ATF-2 [T00167]	NFI/CTF [T00094]
Elk-1 [T00250]	LEF-1 [T02905]	RAR-beta [T00721]	ER-alpha [T00261]
USF2 [T00878]	TBP [T00794]	EBF [T05427]	RAR-beta:RXR-alpha [T05420]
HIF-1 [T01609]	E2F-1 [T01542]	TCF-4 [T02918]	

B

1 gaattcagaa ttttagac**CC** **TTT**ggccttg gggtccatcc tggagaccct gaggtctaag
61 ctacagcccc tcagccaacc acagaccctt ctctggctcc ca**AAAGG**agt tcagtccc**AG**
121 **AGG**gtggtca cccacccttc agggatgaga agttttcaag gggtattact caggcactaa
181 ccccaggaaa gatgacagca cattgccata aagttttggt tgttttctaa gccagtgcaa
241 ctgcttattt tagggatttt ccggg**ATAGG** gtggggaagt ggaaggaatc ggcgagtaga
301 agagaaagcc tgggagggtg gaagttaggg atctagggga agtttggctg atttggggat
361 gcgggtgggg gaggtgctgg atggagttaa gtgaagg**ATA** **GG**gtgcctga gggaggatgc
421 ccgaagtcct cccagaccca cttactcacg gtggcagcgg cgacactcca gtctatcaaa
481 gatccgccgg gatggagagc caggaggcgg gggctgcccc tgaggtagcg gggaggccgg
541 ggggccgggg ggcggacggg acgagtgcaa tattggcggg ggaaaaaaca acactgcacc
601 gcgtcccgtc cctcccgccc gcccgggccc ggatcccgct ccccaccgcc tgaagccggc
661 ccgacccgga acccgggccg ctggggagtt gggttcacct tggaggccag agagacttgg
721 cgcccggaag c**AAAGG**gaat ggcaaggggg agggggagg gagaacggga gtttgcggag
781 tccagaaggc cgctttccga cgcccgggcg ttgcgcgcgc ttgctcttta agtactcaga
841 ctgcgcggcg cgagccgtcc gcatggtgac gcgtgtccca gcaaccgaac tgaatggctg
901 ttgcttggca atgccgggag ttgaggtttg gggccgccca cctagctact cgtgttttct
961 ccggcctgcg agttgggggg ctcccgcctc cccggcccgc tcctgggcgc gctgacgtca
1021 gatgtcccca ccccgcccag cgcctgcccc aagggtctcg ccgcacacaa agctcggcct
1081 cgggcgccgg cgcgcgggcg agagcggtgg tctctcgcct gctgatctga tgcgctccaa
1141 tcccgtgcct cgccgaagtg tttttaaagt gttctttcca a**CCTGT**gtct ttggggctga
1201 gaactgtttt ctgaat**ACAG** **G**cggaactgc ttccgtcggc ct**AGAGG**cac gctgcgactg
1261 cgggacccaa gttccacgtg ctgccgcggc ctgggatagc ttcctcccct cgtgcactgc
1321 tgccgcacac a**CCTCT**tggc tgtcgcgcat tacgcacctc acgtgtgctt ttgccccccg
1381 ctacgtgcct a**CCTGT**cccc aataccactc tgctcccc**AA** **AGG**atagttc tgtgtccgta
1441 aatcccattc tgtcacccca cctactctct gcccccc**CCT** **TT**tttgtttt gagacggagc
1501 tttgctctgt cgcccaggct ggagtgcaat ggcgcgatct cggctcactg caacctccgc
1561 ctcccgggtt caagcgattc tcctgcctca gcctcctgag tagctggggt tacagcgccc
1621 gccaccacgc tcggctaatt tttgtagttt ttagtagaga cgaggtttca ccatcttggc
1681 caggctggtc ttgaacccct gaccttgtga tccactcgcc tcggccttcc aaagtgttgg
1741 gattacgggc gtgacgaccg tgccacgcat ctgcctctta agtacataac ggcccacaca
1801 gaacgtgtcc aactccccg cccacgttcc aacgt**CCTCT** cccacatacc tcggtgcccc
1861 ttccacatac ctcaggaccc cacccgctta gctccatttc ctccagacgc caccaccacg
1921 cgtcccggag tgccccctcc taaagctccc agccgtccac catgctgtgc gttcctccct
1981 ccctggccac ggcagtgacc cttctctccc gggccctgct tc**CCTCT**cgc gggctctgct
2041 gcctcactta ggcagcgctg cccttact**CC** **TCT**ccgcccg gtccgagcgg cccctcagct
2101 tcggcgccca gccccgcaag gctcccggtg accact**AGAG** **G**gcgggagga gctcctggcc
2161 agtggtggag agtggcaagg aaggacccta gggttcatcg gagcccaggt ttactccctt
2221 aagtggaaat ttcttccccc actcctcctt ggctttctcc aaggagggaa cccaggctgc
2281 tggaaagtcc ggctggggcg gggactgtgg gttcagggga gaacggggtg tggaacggg**A**
2341 **CAGG**gagcgg ttagaagggt ggggctattc cgggaagtgg tggggggagg gagcccaaaa
2401 ctagcaccta gtccactcat tatccagc**CC** **TCT**tatttct cggccgctct gcttcagtgg
2461 acccggggag ggcggggaag tggagtggga gacctagggg tgggcttccc gaccttgctg
2521 t**ACAGG**acct cgacctagct ggctttgttc cccatcccca cgttagttgt tgccctgagg
2581 ctaaaactag agcccagggg ccccaagttc cagactgccc ctcccccctc ccccggagcc
2641 agggagtggt tggtg**AAAGG** gggaggccag ctggagaaca aacgggtagt caggggggttg
2701 agcgattaga gcccttgtac cctacccagg aatggttggg gaggaggagg a**AGAGG**tagg
2761 aggtagggga gggggcgggg ttttgtca**CC** **TGT**cacctgc tcgctgtgcc tagggcgggc
2821 gggcggggag tgggggggacc ggtataaagc ggtaggcg**CC** **TGT**gcccgct cca**CCTCT**ca
2881 agcagccagc gcctgcctga atctgttctg ccccctcccc acccatttca ccaccacc**at**
2941 **g**

C

Start position	End position	Dissimilarity	String	RE equally	RE query
19	23	0.207689	CCTTT	11.47656	10.22397
103	107	0.207689	AAAGG	11.47656	10.22397
119	123	0.207689	AGAGG	11.47656	10.22397
266	270	0.000000	ATAGG	11.47656	10.22397
398	402	0.000000	ATAGG	11.47656	10.22397
731	735	0.207689	AAAGG	11.47656	10.22397
1182	1186	0.000000	CCTGT	11.47656	10.22397
1217	1221	0.000000	ACAGG	11.47656	10.22397
1243	1247	0.207689	AGAGG	11.47656	10.22397
1332	1336	0.207689	CCTCT	11.47656	10.22397
1392	1396	0.000000	CCTGT	11.47656	10.22397
1419	1423	0.207689	AAAGG	11.47656	10.22397
1478	1482	0.207689	CCTTT	11.47656	10.22397
1774	1778	0.207689	CCTCT	11.47656	10.22397
1836	1840	0.207689	CCTCT	11.47656	10.22397
2023	2027	0.207689	CCTCT	11.47656	10.22397
2069	2073	0.207689	CCTCT	11.47656	10.22397
2137	2141	0.207689	AGAGG	11.47656	10.22397
2340	2344	0.000000	ACAGG	11.47656	10.22397
2429	2433	0.207689	CCTCT	11.47656	10.22397
2522	2526	0.000000	ACAGG	11.47656	10.22397
2656	2660	0.207689	AAAGG	11.47656	10.22397
2752	2756	0.207689	AGAGG	11.47656	10.22397
2789	2793	0.000000	CCTGT	11.47656	10.22397
2859	2863	0.000000	CCTGT	11.47656	10.22397
2874	2878	0.207689	CCTCT	11.47656	10.22397

Figure S4. Analysis of the promoter sequence of *human MUC1* gene

A) Transcription factors predicted to have binding sites in the *MUC1* promoter, identified with the software PROMO (ALGGEN Research Software). The dissimilarity threshold was lowered to 5% (95% of similarity). Glucocorticoid receptor-alpha (GR-alpha, purple box) was chosen for the identification of putative binding sites in the *MUC1* promoter. **B**) Sequence of the promoter region of human *MUC1* gene (accession number X69118.1) submitted to PROMO. Bolded capital letters mark putative binding sites of GR-alpha. The last three nucleotides identify the ATG start codon. **C**) Features of each GR-alpha binding site from the PROMO output: the *start* and *end* positions of the putative binding sequences are reported; *dissimilarity* indicates the percentage of dissimilarity between the putative and consensus binding sequences; *string* indicates the nucleotide sequence of the potential binding site; *Random expectation (RE)* indicates the expected occurrences of the match in a random sequence of the same length as the query sequence (*RE equally*: equiprobability for the 4 nucleotides; *RE query*: nucleotide frequencies as in the query sequence).

A

Factors predicted within a dissimilarity margin less or equal than 15 % :

Pax-5 [T00070]	p53 [T00671]	GR-alpha [T00337]	PPAR-alpha:RXR-alpha [T05221]
AP-2alphaA [T00035]	FOXP3 [T04280]	PR B [T00696]	PR A [T01661]
ENKTF-1 [T00255]	NF1/CTF [T00094]	GR [T05076]	c-Myb [T00137]
EBF [T05427]	YY1 [T00915]	ER-alpha [T00261]	c-Jun [T00133]
VDR [T00885]	PXR-1:RXR-alpha [T05671]	TBP [T00794]	RXR-alpha [T01345]
HOXD10 [T01425]	HNF-1B [T01950]	USF2 [T00878]	ATF3 [T01313]
HNF-3alpha [T02512]	NF-AT1 [T00550]	AhR:Arnt [T05394]	AhR [T01795]
Egr-3 [T00243]	NF-kappaB1 [T00593]	IRF-2 [T01491]	HNF-4alpha [T03828]
RAR-beta:RXR-alpha [T05420]	PU.1 [T02068]	AP-1 [T00029]	c-Fos [T00123]
RBP-Jkappa [T01616]	MAZ [T00490]	Sp1 [T00759]	NF-kappaB [T00590]
C/EBPbeta [T00581]	C/EBPalpha [T00105]	LEF-1 [T02905]	NF-1 [T00539]
GR-beta [T01920]	TFII-I [T00824]	STAT4 [T01577]	c-Ets-1 [T00112]
HNF-1A [T00368]	RelA [T00594]	XBP-1 [T00902]	GCF [T00320]
TFIID [T00820]	PEA3 [T00685]	Elk-1 [T00250]	AR [T00040]
RAR-beta [T00721]	GATA-1 [T00306]	HNF-1C [T01951]	HOXD9 [T01424]
c-Ets-2 [T00113]	T3R-beta1 [T00851]	RAR-alpha1 [T00719]	IRF-1 [T00423]
SRY [T00997]	TCF-4E [T02878]	GATA-2 [T00308]	E2F-1 [T01542]
COUP-TF1 [T00149]	STAT5A [T04683]	Ik-1 [T02702]	MEF-2A [T01005]
NF-AT2 [T01945]	STAT1beta [T01573]	NF-AT1 [T01948]	ETF [T00270]
NF-Y [T00150]	E2F [T00221]	TCF-4 [T02918]	

B

```
   1 gtcgtgggcg gtcggcccag gtgattgccc ttcttttgct ttggagccag gcagaacaga
  61 gctcaaattc cagctttggg acagtgtgtg gcccaaaaag ctgtaactgc ggagttaaca
 121 ctctccagtt tccctagatg gtccgacctg gccggtgact cggggatgat gaagctcctg
 181 gagcctcagc gtgctcactt gagaggtagg gcagcgcttt gtacttcaca gggccatggg
 241 gacaggctca gggcctaaaa ctctagtgac cggcttcatg ctgggtcact gactagggaa
 301 tccacacacc tttttagtcc agactgagta ggtaaatcag acttaataaa catccagctt
 361 cctgctcgag ctcagcctag ttgtcctaag tctgacaaga ttgaacttta ttcacagcac
 421 gaggcataaa tgatataaag gtaagcaatt ttaggggacc caaaaaacta gggactccta
 481 cttgggtagc ttagaagggg aatctctctt ggagatcctg gctttgctgc CTCAAACCCA
 541 gctgtctctt caagccaagt ctgggagaat ctcatgatct tatccttgct ccaatttact
 601 tacaccccac atccacacta agctcttcct gcctcccgtg gaatattaac ttacacctga
 661 cttcagactc ctgcccctat gcagacccag cagtatgcgg gcctaggagg tttttcggcc
 721 acaaggaact agagaagcca ctcattttgt cccctctcat tcaccccatt ctgtccccat
 781 cggatgcctt gggaggagag aaggaaggac aaatgggtGT CCCGTGACCC AACCCAgatg
 841 aagacagagc catttatcac agagacccag acgcatccta actctggaaa atgggcatat
 901 tgaggggagc tggaaagcag aagggaaacg ggcgtgggca gggaagaggt gaacaggagt
 961 gcgcacgccc agttctccag gatctcacgc tcctaatcag gatcctataa gactcaaagg
1021 ggacgcagga aagacctggg cctggaaatt cacttccgga ctcctcctgt atcctggctg
1081 gccctcgttt gtgtctccct ccagggatga tgcccggatg gaaggaggag ccacgttcct
1141 ccacaggtgt cctccacaac aggtgtcctc acaaccaggt gtcgtaacaa caaggtgtcc
1201 tcacaacaag gtgtcctaat acctcccgca gcccctcagc atctacaggc tcactcccac
1261 cctacaacct ttcagatagg tgtatcccca ttttacaggt gaacgaggaa aaaagtgaag
1321 gctgtgactt tgtggtgacg cggtggcaga gctgggatca agccCGGGTT TGTAacgccc
1381 tcctggagtc tcggccgtca cagcagcttc cggagagcgc tgtgtgcctc tggccttcct
1441 ggcatgggaa agaggtcagc tacctgtgtt cggtcaacaa gatgtccagc gcttagtttg
1501 gtctcacctg gatgctcctt tctcctctag agatggaaaa ctgttgtcct gaaagcgtca
1561 ccaaacaaaa cctgtggaaa ggtaatgggg ggacccacga gcaagagctg acagaaaaat
1621 gaccacgggc cgggtgcagt ggctcacgcc tgtaatccca gcactttggg aggccaaggc
1681 gggcggatca tctgagttca GGGGTTCGAG accaacctgg ccaacatggt aaaacctcgt
1741 ccctactaaa aatacaaaaa aaattagccg ggcgtggtgg caggtgcctg tagtcccagc
1801 tactagggag gctgaggcag gggaaTGGCG TGAACCCGgg aggcggagct tgcagtgagc
1861 tgagatggtg ccactgcact ccagcctggg gcgacagagc aagactccat ctcaaaaaaa
1921 aaaaagtgca aaatgatcac gaaggccaag ctgggccttg gtgtctgcga attccctgca
1981 gtaggtccct cctgctgccc cgtccaggcg cccacaggat gctccacatg actctttacc
2041 tttatgattt tcttacctcc aggaaagaac agggtcagct cgtccaccca gagctggagg
2101 gactgttggg ggaccacgcc acttccccac ggtatccgca ttatcatggc gtggcccagg
2161 ctttggcctg ggccatgaac agactttcag cttctagaag caagtggtca cagggCCTCC
2221 GTGACCCTcg gagacagcgc cacagcgtcg gttgcctact ccttgctgga caaggctggc
2281 tccgcttggg aatgCAGAAA CCCTgagtcc ttgtcctgga ccccacagtg tctgagcatc
2341 gaaggtccct gctctgggac gagtcacccc acagacaccc cacattcggg aatcaagtct
2401 tgcggaatca gaagcagaga ttcacactgt ggcaaagagg ggagcggagg tatccccgtt
2461 ttcctttgca gagagctaca ccaactgccc tcttcgtctT TACAACCCTa cttggggtcc
2521 ctttccctcc tcgcttgtgc ctgtgagttc caagaggtgg aactggggT GAGCAGAGCC
2581 Ccccaggtct ccaggagcag ctccacctcc tcacaggtta cagtcgcttc ctcctaccga
2641 ggttttgcCT TCAACCCAct gcccgtgcca gctccctcag gccagtctcc cagactgtcc
2701 ctggctccct gcaccctggc tccagctccc tgccctggaa agaagaagtc caccatctgg
2761 tgagggtctg ccacgcatct ggggtctgcc atgcgtctgc actgaggttc tgccacgagt
2821 ctgcactgag gttctgccat gcgtctggag tctgccacgc gtctggggtc tgccacgcgt
2881 ctggagtctg ccatgggtct ggggtctgcc atgtgtctgg gatctgccgt gcgtctgggg
2941 tctgccacgc atctgcactg aggttctgcc acgcgtctgg ggtctgccat gcgtctgcac
3001 tgaggttctg ccacgcgtct ggggtccgcc atgcgtctgg ggtctgccac gcgtctgggg
3061 tctgccacgc acctgcactg agcagatatt ccaagcacac caccactttc caaaagacgg
3121 catacagtgc atttctgttc ctgcccctca cccacatggt tccccgcttc ccaattcctt
3181 ggggtctgct taagtctcac tctctttccc ccattcatac agccccaagg tcgctccctc
3241 tggggccctt tcttccccat tcttcccagc agcccaaagc tctggtggga caggggcagc
3301 ccctggggag ggaggagagg accCAGGAAC CCGgctagga gggtggccca cccatttcca
3361 gtgTGACCTG TTCCCattcc cccatgtctc ctcccatccc tcccgccact cagctcaggc
3421 tgatgagaag cagagcaacg ggtgtatcgg tgttttcttt cctggtgggg tagtggggtg
3481 gggctgagga gagaaaaggg tgattagcgt ggggccccgc cctcttttgt cctctTCCCA
3541 GGTTCCCTGG CCCCttcgga gaaacgcact tggttcgggc cagccgcctg aggggacggg
3601 ctcacgtctg ctcctcacac tgcagctgct gggccgtgga gcttccccag ggagccaggg
3661 ggacttttgc cgcagccatg
```

BLACK bold: RAR-beta:RXR-alpha
BLUE bold: RAR-alpha
BROWN bold: RAR-beta
shaded sequences: shared by overlapping binding sites

C

Start position	End position	Dissimilarity	String	RE equally	RE query
RAR-beta:RXR alpha					
1826	1837	14.955991	TGGCGTGAACCC	0.38561	059539
2570	2581	12.463326	TGAGCAGAGCCC	0.24084	0.37816
3364	3375	14.955991	TGACCTGTTCCC	0.38561	059539
3536	3547	14.955991	TCCCAGGTTCCC	0.38561	059539
3543	3554	12.463326	TTCCCTGGCCCC		
RAR-alpha					
819	831	13.441672	GTCCCGTGACCCA	0.05736	0.07299
2216	2228	11.143532	CCTCCGTGACCCT	0.03032	0.03582
RAR-beta					
531	540	3.244529	CTCAAACCCA	0.22461	0.24826
827	836	7.478240	ACCCAACCCA	0.44922	0.49744
1365	1374	6.452127	CGGGTTTGTA	033691	0.34136
1701	1710	4.326039	GGGGTTCGAG	0.26672	0.28233
1829	1838	5.389083	CGTGAACCCG	0.28076	0.32228
2295	2304	8.541284	CAGAAACCCT	0.49133	0.52651
2500	2509	10.667372	TTACAACCCT	0.25269	0.26114
2649	2658	7.496706	CTTCAACCCA	0.44922	0.49744
3324	3333	10.704304	CAGGAACCCG	0.25269	0.26114

Figure S5. Analysis of the promoter sequence of *human MUC4* gene

A) Transcription factors predicted to have binding sites in the *MUC4* promoter, identified with the software PROMO (ALGGEN Research Software). The dissimilarity threshold was left at the default value of 15% (85% of similarity). RAR-beta:RXR-alpha (black box), RAR alpha (blue box), and RAR-beta (red box)were chosen for the identification of their putative binding sites in the *MUC4* promoter. **B**) Sequence of the promoter region of the human *MUC4* gene (accession number AF241535.2) submitted to PROMO. Bolded capital letters mark the putative binding sites for RAR-beta:RXR-alpha (black), RAR alpha (blue), and RAR-beta (brown). Overlapping binding sites are shaded. The last three nucleotides identify the ATG start codon (red). **C**) Features of each transcription factor binding site: the *start* and *end* positions of the putative binding sequences are reported; *dissimilarity* indicates the percentage of dissimilarity between the putative and consensus binding sequences; *string* indicates the nucleotide sequence of the potential binding site; *Random expectation (RE)* indicates the expected occurrences of the match in a random sequence of the same length as the query sequence (*RE equally*: equiprobability for the 4 nucleotides; *RE query*: nucleotide frequencies as in the query sequence).

```
   1 gaccaagccc agttcccaat tggccctttt gcccggggag gtccttagtg gagggagagg
  61 gcaaagtcgg gcccccaatt gtttggtgtc actgtgtgcc agcgggccac tggcgggcga
 121 ggttgttcca gggtggaggc ggggagggtt ggaccacagg cactgagcgg ggacagagga
 181 gctgcctgag ggtcccagct ctgccatgga gaaaacgcta tctcgctgat gcagaggtgc
 241 ccggcccact cgagctgggg gtgagggggc tgctccccag tgggccgcca gccccatga
 301 aggccgcggg caccggccgt ggtcagggag ggcaggggac aggcagtggg ggccagcagg
 361 ggagacacta ggcttggccc cagcacccag gtgggcatcg gcttgtgagc tggagccgcg
 421 ggcagggagg ggggatgtca cgagggcttg gctaaggtgg gagacctggg cgggtgcgtc
 481 gggggacgtc tgcagcagag gcctgggcag caggcacacc cctcctgcca gtgcgaggaa
 541 cgaggcgcca cagcggccgg tagcccccca tttgcccaGC CTGGCctgga gcaggcagga
 601 aggccgggga gaggggtctg gctggggcct gggtgcagtc acagccacga gcccaggggt
 661 ggggagtctg gcccaccctc cagaccatcc tcaaggccca ctggcccagg catccccgcc
 721 cacccctccc accgtgccgt gctgcagcgg gtctaccggc ctggatgtga aagagagctt
 781 ggagaccccca gagacctcgg aaccttcagc tttggaagtg acgtcggtgg ggtgggtggg
 841 gggggcacag gctctggagt cccggaagtg agcggggagc tacgctgaga tctgggagac
 901 cccctgcccc cacccaggta cagggccagg cagaagcccg aggtgtgccc tgaggtaaag
 961 aaaccgtcac aaagaacaaa gggagaagGC gGGttCCAgc ctccaccaca gccctcgcgc
1021 tctgaggagc cacctggggg cctcagccat gaggggtgac aggtggcaaa acgggccagc
1081 tccgttcacg tcgctgtgca gctgtctccg ccctccatct ccagaacgtt ctcacattcc
1141 caagctgaaa ccctgtcccc atgcaacacc agctcaccat cccctctgcc agcccctggc
1201 gcccaccgtc cacactccgt ctctgcgggt ttcatgactc caggggcagc acacgagtgg
1261 cccctcctgc ctttgtcctc tgtgtccacc tgcctcactc tgcacagtgt ccccagcttc
1321 ccccatggag cagcctgggc cagcctctcc ttttcacggc tgaaccgtat tccaccgcac
1381 ggatcagcct cacgatgctg acccagtcct ccgcccaggg acacatgggc agcttctgcc
1441 tttgtcagtg atgctgctgt ggacatgggt gtgcaaatgt ccctcaggac ccgccttcag
1501 ttcttctggg gacagaccca gagtggagtt gctggtcacc cccaccagca gggcacaggg
1561 ctccgggtcc ccacgtctct gccaacactt cctacttcct gtgtttcttg atccccgcca
1621 tcctattgag cgtgagacag gtcagaagct ttgaagatgg gctttcgtct tgtcccagaa
1681 atcccacctc taagaattta acttcagaaa gacaaacgcg gggagctgg tgcagggccc
1741 gtgacgggga ctgtgacgta aataaaacaa cagacctgga caccacccta gggtccccat
1801 ggggccggac gaggccacac cacccgacct ggtgcttcca tGCCTGGCgt ctgcgccacg
1861 gagcatttca ggacgctggt gaccagggag ccaggaggtg ggagcatctg aggtgcaggt
1921 cacacgggca ggaggtgttt gcaagaggta ttgcagcgcg gacggagtgt cctgcagatg
1981 acgctgtctg tcctgtagat gacgctcgtc aaggaggttt accacatagc ccccaggaag
2041 cccacccgac accagccgga ggtgctaggc ttctgcggct cccacctggg gcaggcggag
2101 gaccccgggc aggtccagga ccccccggag cagctgcttc ctcaaccctg ccagggttaa
2161 tgaggaggcc ccagagtgag gtggaggcca aatgggactc agggccggag cctctgGCCT
2221 GGCtggatca gggctggcat tggacaagcg cagctgactc ccgatgtgca tggccaggag
2281 acactctggg cctcagtctc cccttgaatg tgaaccttga aacagatcag cccagagacc
2341 tcccacggtc ttcaaggggc tctggtcagc tgggctgggg tctctggaaa tagagcctct
2401 tccagggacc cccacaagcc acccagactg agcatcctgg ccatgtgcat gcctgagctc
2461 cagcctcgtg ggccctcccc gggcctcggg gacccatggt cagtggctgg gggtgctgcc
2521 cagaggctgg gattcccttc cagcaggagc cgcagtgggg ctgagtgtga ggcaggctgg
2581 ctgaccactg tttccatgga ccctgcgtcc aaggccagcc ctgccttcca gcggctttgc
2641 catctaggac gggtgccagg tggggtaggc ccttctctcc cttccgattc tcagaagctg
2701 ctggggggtgg gggcgtcctg ggcctcaggg cacagagctg caaatccttc ctgatccagg
2761 cctctcccct gccacagccc ctccccgaga gcaaacacac gtggctggag cggggaagag
2821 cacggtgccc tgcgtgGCCT GGCCTGGCtt ggggccaagg ctccctgcta cataagctgg
2881 ggcccccagg ggagcaagca cccggcccgg ctccctccct gcccgtcccc gtccccccac
2941 ccgtgccagc ccccaggatg
```

Figure S6. ***MUC5B*** **promoter sequence (accession number AJ012453)**
Green bold capital letters mark the butyrate response elements [37]. The nucleotide sequence high-lighted in blue matches the consensus sequence of butyrate response elements (**GCgGGctCCA**) [38]. Exact matches were retrieved using BLASTn search (NCBI). The last three nucleotides identify the ATG start codon (red).

Table S1. Mucin expression pattern in control tissue (normal gut mucosa) of Colon and Ileum
Expression in cellular compartments is classified as: **c**, cytoplasmic (non-vacuolar); **m**, membranous (apical membrane); and **v**, vacuolar (glicocalix). N.D., not detected.

Case n.	*Tissue*	*MUC1*	*MUC2*	*MUC4*	*MUC5B*	*MUC12*	*MUC13*	*MUC15*	*MUC17*
1	Colon	v+m	v	c+v	c+v	m	c	m	N.D.
2	Colon	v+m	v	c+v	c+v	m	c	m	N.D.
3	Colon	v+m	v	c+v	c+v	m	c	m	N.D.
4	Colon	v+m	v	c+v	c+v	m	c	m	N.D.
5	Colon	v+m	v	c+v	c+v	m	c	m	N.D.
6	Colon	v+m	v	c+v	c+v	m	c	m	N.D.
7	Colon	v+m	v	c+v	c+v	m	c	m	N.D.
8	Colon	v+m	v	c+v	c+v	m	c	m	N.D.
9	Colon	v+m	v	c+v	c+v	m	c	m	N.D.
10	Colon	v+m	v	c+v	c+v	m	c	m	N.D.
1	Ileum	N.D.	v	N.D.	c	N.D.	c+m	N.D.	m
2	Ileum	N.D.	v	N.D.	c	N.D.	c+m	N.D.	m
3	Ileum	N.D.	v	N.D.	c	N.D.	c+m	N.D.	m
4	Ileum	N.D.	v	N.D.	c	N.D.	c+m	N.D.	m
5	Ileum	N.D.	v	N.D.	c	N.D.	c+m	N.D.	m
6	Ileum	N.D.	v	N.D.	c	N.D.	c+m	N.D.	m
7	Ileum	N.D.	v	N.D.	c	N.D.	c+m	N.D.	m
8	Ileum	N.D.	v	N.D.	c	N.D.	c+m	N.D.	m
9	Ileum	N.D.	v	N.D.	c	N.D.	c+m	N.D.	m
10	Ileum	N.D.	v	N.D.	c	N.D.	c+m	N.D.	m

Table S2. Mucin expression pattern in treatment-naïve UC patients

Expression in cellular compartments is classified as: **c**, cytoplasmic (non-vacuolar); **m**, membranous (apical membrane); and **v**, vacuolar (glicocalix). N.D., not detected.

Case n.	*MUC1*	*MUC2*	*MUC4*	*MUC5B*	*MUC12*	*MUC13*	*MUC15*	*MUC17*
**1**	c+v+m	v	c	c	m	c	m	N.D.
**2**	c+v+m	v	c	c+v	m	c	m	N.D.
**3**	c+v+m	v	c	c	m	c	m	N.D.
**4**	c+v+m	v	c	c	m	c	N.D.	N.D.
**5**	c+v+m	v	c	c	m	c	N.D.	N.D.
**6**	c+v+m	v	c	c	m	c	N.D.	N.D.
**7**	c+v+m	v	c	c	m	c	N.D.	N.D.
**8**	c+v+m	v	c	c+v	m	c	m	N.D.
**9**	c+v+m	v	c	c	m	c	m	N.D.
**10**	c+v+m	v	c	c	N.D.	c	N.D.	N.D.
**11**	c+v+m	v	c	c+v	m	c	N.D.	c
**12**	c+v+m	v	c	c	m	c	N.D.	N.D.
**13**	c+v+m	v	c	c+v	m	c	N.D.	N.D.
**14**	c+v+m	v	c	c	m	c	N.D.	N.D.
**15**	c+v+m	v	c	c	m	c	m	N.D.
**16**	c+v+m	v	c	c+v	m	c	m	N.D.
**17**	c+v+m	v	c	c+v	m	c	m	N.D.
**18**	c+v+m	v	c	c	m	c	N.D.	c
**19**	c+v+m	v	c	c	m	c	N.D.	N.D.
**20**	c+v+m	v	c	c	m+c	c	m	N.D.
**21**	c+v+m	v	c	c	m	c	m	c
**22**	c+v+m	v	c	c+v	m	c	N.D.	c
**23**	c+v+m	v	c	c	m+c	c	m	c
**24**	c+v+m	v	c	c+v	m	c	N.D.	N.D.
**25**	c+v+m	v	c	c	m	c	m	c
**26**	c+v+m	v	c	c	m	c	m	N.D.
**27**	c+v+m	v	c	c	m	c	N.D.	N.D.
**28**	c+v+m	v	c	c+v	m	c	m	N.D.
**29**	c+v+m	v	c	c	m	c	m	N.D.
**30**	c+v+m	v	c	c	m	c	N.D.	N.D.
**31**	c+m	v	c	c+v	m	c	m	N.D.
**32**	c+m	v	c	c+v	m	c	N.D.	N.D.
**33**	c+v+m	v	c	c+v	m	c	m	N.D.
**34**	c+m	v	c	c	m	c	m	N.D.
**35**	c+v+m	v	c	c	m	c	N.D.	N.D.
**36**	c+m	v	c	c+v	m	c	m	N.D.
**37**	c+m	v	c	c	m	c	m	N.D.
**38**	c+v+m	v	c	c	m	c	m	c
**39**	c+m	v	c	c+v	m	c	m	N.D.
**40**	c+m	v	c	c+v	m	c	m	N.D.

Red: abnormal expression pattern.

Table S3. Mucin expression pattern in UC patients after first-line treatment

Expression in cellular compartments is classified as: **c**, cytoplasmic (non-vacuolar); **m**, membranous (apical membrane); and **v**, vacuolar (glicocalix).

Case n.	*Clinical state*	*Terapy*	*MUC1*	*MUC2*	*MUC4*	*MUC5 B*	*MUC1 2*	*MUC 13*	*MUC 15*	*MUC 17*
**1**	Relapse	NSAIDs	c+m	v	c	c+v	m	c	m	//
**2**	Relapse	Biol. Ag	c+m	v	c	c+v	m	c	m	c
**3**	Relapse	NSAIDs,GC	c+m	v	c	c+v	m	c	//	//
**4**	Relapse	NSAIDs	c+v+m	v	c	c+v	m	c	m	//
**5**	Relapse	NSAIDs	v+m	v	c	c+v	m	c	//	c
**6**	Relapse	Biol. Ag	c+m	v	c	c+v	m	c	m	//
**7**	Relapse	Biol. Ag	c+m	v	c	c+v	m	c	m	//
**8**	Relapse	NSAIDs	c+m	v	c	c	m	c	//	//
**9**	Relapse	NSAIDs	c+m	v	c	c	//	c	m	//
**10**	Relapse	Biol. Ag	c+m	v	c	c+v	m	c	m	c
**11**	Relapse	NSAIDs	c+m	v	c	c	m	c	m	//
**12**	Relapse	Biol. Ag	c+m	v	c	c+v	m	c	//	//
**13**	Relapse	Biol. Ag	c+v+m	v	c	c	m	c	m	//
**14**	Relapse	Biol. Ag	c+v+m	v	c	c	m	c	//	//
**15**	Relapse	NSAIDs	c+m	v	c	c	m	c	m	c
**16**	Relapse	GC	c+m	v	c	c+v	m	c	m	//
**17**	Relapse	GC	c+m	v	c	c+v	m	c	//	//
**18**	Relapse	NSAIDs	c+v+m	v	c	c+v	m	c	m	//
**19**	Relapse	GC	c+m	v	c	c	m	c	m	//
**20**	Relapse	GC	c+v+m	v	c	c	m	c	//	//
**21**	Remission	GC	v+m	v	c	c+v	m	c	m	//
**22**	Remission	GC	v+m	v	c	c	m	c	m	//
**23**	Remission	NSAIDs	c+v	v	c	c	m	c	m	//
**24**	Remission	NSAIDs	c+v	v	c	c	m	c	m	//
**25**	Remission	NSAIDs	c+v	v	c	c+v	m	c	m	//
**26**	Remission	NSAIDs	c+v	v	c+v	c+v	m	c	m	//
**27**	Remission	NSAIDs	c+v	v	c	c+v	m	c	m	//
**28**	Remission	Biol. Ag	c+m	v	c	c	m	c	m	//
**29**	Remission	NSAIDs	c+v	v	c	c+v	m	c	m	//
**30**	Remission	NSAIDs	c+v	v	c	c+v	m	c	m	//
**31**	Remission	NSAIDs,GC	v+m	v	c	c+v	m	c	m	//
**32**	Remission	NSAIDs	c+v	v	c	c	m	c	m	//
**33**	Remission	NSAIDs	c+v	v	c	c	m	c	m	c
**34**	Remission	GC	v+m	v	c	c+v	m	c	m	//
**35**	Remission	Biol. Ag	c+v	v	c	//	m	c	m	//
**36**	Remission	Biol. Ag	c+v	v	c+v	c+v	m	c	m	//
**37**	Remission	NSAIDs	c+v	v	c+m	c	//	c	//	c
**38**	Remission	NSAIDs	c+v	v	c	c	//	c	//	c
**39**	Remission	NSAIDs,GC	v+m	v	c	c	//	c	//	c
**40**	Remission	NSAIDs,GC	v+m	v	c	c	m	c	m	//

Red: abnormal expression pattern.
NSAIDs: nonsteroidal anti-inflammatory drugs. **GC**: glucocorticoids. **Biol. Ag**.: biological agents

Table S4. Anti-mucin antibodies used in the study

Mucin	Vendor (catalog no.)	Host species-Clonality	Reactivity	Dilution	Antigen retrieval
**MUC1***	Abcam (ab109185)	Rabbit - Monoclonal	Human	1:250	Citrate buffer
**MUC2***	Abcam (ab134119)	Rabbit - Monoclonal	Human	1:800	Citrate buffer
**MUC4***	Abcam (ab150381)	Rabbit - Monoclonal	Human	1:750	Citrate buffer
**MUC5B***	Abcam (ab87376)	Rabbit - Polyclonal	Human	1:400	Citrate buffer
**MUC12***	Abcam (ab121777)	Rabbit - Polyclonal	Human	1:250	EDTA
**MUC13***	Abcam (ab235450)	Rabbit - Polyclonal	Human	1:4000	EDTA
**MUC15***	Abcam (ab224468)	Rabbit - Monoclonal	Human	1:500	EDTA
**MUC15****	Invitrogen (BS-5878R)	Rabbit - Polyclonal	Human	1:100	Citrate buffer
**MUC17***	Abcam (ab122184)	Rabbit - Polyclonal	Human	1:200	EDTA

*IHC staining **IF staining
